# Environmental persistence of porcine coronaviruses in feed and feed ingredients

**DOI:** 10.1371/journal.pone.0178094

**Published:** 2017-05-24

**Authors:** Michaela P. Trudeau, Harsha Verma, Fernando Sampedro, Pedro E. Urriola, Gerald C. Shurson, Sagar M. Goyal

**Affiliations:** 1 Department of Animal Science, University of Minnesota, St. Paul, Minnesota, United States of America; 2 Veterinary Population Medicine, University of Minnesota, St. Paul, Minnesota, United States of America; Universidade de Aveiro, PORTUGAL

## Abstract

Porcine Epidemic Diarrhea Virus (PEDV), Porcine Delta Corona Virus (PDCoV), and Transmissible Gastroenteritis Virus (TGEV) are major threats to swine health and contaminated feed plays a role in virus transmission. The objective of our study was to characterize inactivation of PEDV, PDCoV, and TGEV in various feed ingredient matrices. Samples of complete feed, spray dried porcine plasma, meat meal, meat and bone meal, blood meal, corn, soybean meal, and corn dried distillers grains with solubles were weighed (5 g/sample) into scintillation vials and inoculated with 1 mL of PEDV, PDCoV, or TGEV. Samples were incubated at room temperature for up to 56 days. Aliquots were removed at various time points followed by preparing serial 10-fold dilutions and inoculating in cell cultures to determine the amount of surviving virus. Inactivation kinetics were determined using the Weibull model, which estimates a delta value indicating the time necessary to reduce virus concentration by 1 log. Delta values of various ingredients were compared and analyzed as to their nutrient composition. Soybean meal had the greatest delta value (7.50 days) for PEDV (*P* < 0.06) as compared with all other ingredients. High delta values (*P* < 0.001) were observed in soybean meal for PDCoV (42.04 days) and TGEV (42.00 days). There was a moderate correlation between moisture content and the delta value for PDCoV (r = 0.49, *P* = 0.01) and TGEV (r = 0.41, *P* = 0.02). There was also a moderate negative correlation between TGEV survival and ether extract content (r = -0.51, *P* = 0.01). In conclusion, these results indicate that the first log reduction of PDCoV and TGEV takes the greatest amount of time in soybean meal. In addition to this, moisture and ether content appear to be an important determinant of virus survival in feed ingredients.

## Introduction

Coronaviruses belong to the order *Nidovirales*, family *Coronaviridae*, and subfamily *Coronavirinae* [1], and are characterized by their large genome, helical nucleocapsids, and unique method of gene expression [2]. The subfamily includes four genera: *Alphacoronavirus*, *Betacoronavirus*, *Gammacoronavirus*, and the more recently discovered *Deltacoronavirus* [3]. Coronaviruses are typically species specific and can infect a variety of birds and mammals [2]. In humans, coronaviruses are typically responsible for upper respiratory infections and are the second leading cause of the common cold [2]. In swine, there are 3 coronaviruses (Transmissible Gastroenteritis Virus, TGEV; Porcine Epidemic Diarrhea Virus, PEDV; Porcine Delta Corona Virus, PDCoV) that affect gastrointestinal health [4,5].

Transmissible Gastroenteritis Virus has been identified as a cause of severe diarrhea, dehydration, vomiting, and high mortality in neonatal pigs since the 1960’s [6]. The first case of PEDV occurred in the United States in 2013 and led to a devastating outbreak with a high mortality in piglets observed in 17 different states [7]. Shortly after the PEDV outbreak, cases of Porcine Delta Corona Virus (PDCoV) were identified in the United States in 2014 [3]. The signs of disease in PDCoV infected pigs are similar, but less severe than in pigs infected with PEDV and TGEV [7].

Until recent years, the main concern involving feed safety has been the risk of contamination with *Salmonella*. Several research studies have evaluated the survivability of *Salmonella* in feed, along with the effect of processing on reducing its concentration in feed [8–13]. During the initial PEDV outbreak, batches of feed containing spray-dried porcine plasma were found to be PCR positive for the virus, indicating that viruses may be transmitted through feed [14,15]. Subsequent research confirmed that feed contaminated with PEDV was capable of causing an active infection in pigs after consuming it [16]. With the high concentrations of PEDV excreted in feces of infected pigs, only 1 gram of infected feces is necessary to contaminate over 450,000 kilograms of feed [17]. Different heat treatments, such as spray drying, and some feed additives are capable of reducing PEDV concentration in feed and feed ingredients [18–21]. The results from these studies suggest that virus contamination likely occurs post-processing in order to cause an active infection in pigs. If contamination does occur after processing, multiple feed ingredients may be at risk to post-processing contamination. This risk was confirmed by a risk assessment analysis was conducted by researchers at the University of Minnesota that showed the risk of PEDV surviving thermal processing of porcine by-products was negligible, but in some facilities, the risk of post-processing contamination was low to moderate [22].

With the potential risk of post-processing contamination, feed mills are developing biosecurity plans to minimize the risk of corona virus transmission from feed, feed ingredients, and feed transport. To do this, identifying ingredients that are of greater risk of prolonging the survival of these corona viruses is essential for developing these biosecurity procedures. Previous research studies have shown that PEDV survival in feed varies depending on the specific ingredient, with soybean meal appearing to promote extended survival time [23]. However, there has been limited research published that has determined survival of TGEV and PDCoV in feed and feed ingredients [24,25]. Therefore, the objective of this study was to characterize the survival of PEDV, TGEV, and PDCoV in complete feed and feed ingredients. We hypothesized that the three corona viruses would have a similar inactivation pattern in all ingredients evaluated and that chemical composition may be a contributing factor to virus survival.

## Materials and methods

### Virus propagation

The NVSL (National Veterinary Services Laboratory, Ames, IA) strain of PEDV was propagated in Vero-81 (African green monkey kidney, ATCC, CCL-81^™^) cells. The NVSL strain of PDCoV and Purdue strain of TGEV (Purdue University, West Lafayette, IN) strains were propagated and titrated in ST (swine testicular) cells. The cells were grown in Minimum Essential Medium (MEM) with Earle’s salts supplemented with L-glutamine (Mediatech, Herndon, VA), 8% fetal bovine serum (FBS; Hyclone, South Logan, UT), 50 μg/mL gentamicin (Mediatech), 150 μg/mL neomycin sulfate (Sigma, St. Louis, MO), 1.5 μg/mL fungizone (Sigma), and 455 μg/mL streptomycin (Sigma). The maintenance medium for PEDV included Dulbecco's Modified Eagle Medium (DMEM, Mediatech) with antibiotics, and 10 μg/mL of trypsin (Gibco, Life Technologies, Grand Island, NY). For PDCoV, the maintenance medium consisted of MEM with antibiotics and 5 μg/mL trypsin (Gibco). Maintenance medium for TGEV consisted of MEM with antibiotics and 4% donor horse serum (DHS, Hyclone). The cells were washed thrice with phosphate buffered saline (PBS, pH 7.2) before virus inoculation. After virus inoculation, the cells were incubated at 37°C for 1 h for virus adsorption using appropriate maintenance medium. Inoculated cells were incubated at 37°C under 5% CO_2_ for up to 8 days. The inoculated cells were observed for the appearance of virus-induced cytopathic effects (CPE). The CPE appeared at about 8 to 10 days post-infection for PEDV, and 5 to 6 days post-infection for PDCoV and TGEV. The cells were subjected to three freeze-thaw cycles (-80°C/25°C) followed by centrifugation at 2500 × g for 15 min at 4°C. The supernatant was collected and aliquoted into 50 mL centrifuge tubes (Corning, Life Sciences, NY) followed by storage at -80°C until used.

### Feed and feed ingredient samples and composition

The complete feed used in the experiment was obtained from the University of Minnesota College of Veterinary Medicine swine isolation barns, and was obtained from Vita-Plus (CGI enhanced phase II starter feed; Madison, WI). This diet did not contain any animal by-products (batch no. 831458). The feed ingredients including spray dried porcine plasma (SDPP), meat meal, meat and bone meal, blood meal, corn, soybean meal, as well as low, medium and high oil corn dried distillers grains with solubles, were obtained from the University of Minnesota feed mill at the Southern Research and Outreach Center (Waseca, MN). Samples of feed and feed ingredient were submitted to the Minnesota Valley Testing Laboratory (New Ulm, MN) for chemical composition analysis (Table 1). Standard AOAC procedures were used to determine moisture (method 930.15), ash (method 942.05), ether extract (method 2003.05), crude fiber (method 930.39), and crude protein (method 990.03) content [26]. The pH of feed and feed ingredients was measured by mixing 50 mL of distilled water with 5g of sample. The mixture was then stirred on a magnetic stirrer for 20 min to allow the feed to be suspended in the liquid. The pH of the suspension was then measured using a pH probe (Fisher Scientific, Waltham, MA) and the value was recorded. The pH was measured in triplicate, while chemical composition values were determined from a single measurement.

**Table 1:** Analyzed chemical composition of feed ingredients.

Ingredient	Moisture (%)	Ash (%)	Ether extract (%)	Crude fiber (%)	Crude Protein^1^ (%)	pH^2^
Complete Feed	8.57	9.45	4.47	2.02	24.20	5.82 ± 0.02
Soybean Meal	12.12	6.42	0.71	3.26	45.40	6.73 ± 0.01
Corn	14.90	1.55	3.86	1.55	7.03	6.21 ± 0.06
Low Oil DDGS	10.40	30.70	5.87	5.77	4.56	4.31 ± 0.03
Medium Oil DDGS	10.38	29.91	9.85	5.16	4.03	3.81 ± 0.03
High Oil DDGS	9.66	28.57	14.23	5.41	4.62	4.17 ± 0.04
Vitamin-trace mineral premix	2.41	73.77	1.42	1.62	1.91	3.49 ± 0.03
Spray Dried Porcine Plasma	11.60	7.44	0.15	0.01	77.79	7.15 ± 0.02
Blood Meal	11.58	1.79	0.16	0.05	92.60	8.40 ± 0.11
Meat Meal	4.80	24.26	13.54	1.83	54.90	6.64 ± 0.04
Meat and Bone Meal	5.74	24.77	10.77	1.16	55.70	6.50 ± 0.01

^1^Crude protein was calculated from nitrogen content × 6.25.

^2^Average of 3 replicates.

### Virus survival in feed and feed ingredients

All feed samples tested negative for PEDV, PDCoV and TGEV using real time RT-PCR. Five gram aliquots of complete feed and feed ingredients (plasma, meat meal, meat and bone meal, blood meal, corn, soybean meal, as well as low, medium and high oil corn dried distillers grains with solubles) were prepared in scintillation vials. One mL of either PEDV, PDCoV, or TGEV with initial titers of 3.2 × 10^4^, 1.5 × 10^6^, 6.8 × 10^6^, respectively, was added to the appropriate vials and mixed thoroughly. Only one virus was added to each ingredient sample to prevent interactions between viruses in a single ingredient sample. All samples were stored at room temperature (approximately 25°C) for 0, 1, 3, 7, 14, 21, 28, 35, 42, 49, and 56 days. The samples were stored uncovered and the humidity in the room was not controlled. This experiment was performed in triplicate with each of the viruses to produce three replicates for PEDV, PDCoV, and TGEV.

### Virus elution

In all experiments, the surviving virus was recovered in an eluent consisting of a 3% solution of beef extract in 0.05M glycine (pH of 7.5). The elution buffer was the same for each of the three viruses evaluated. The elution procedure has been used in previous experiments with calicivirus and was modified to be used in feed samples [27]. The same modified method was used in previous experiments with PEDV and PDCoV in complete swine feed, but this modified method has never been used to elute TGEV from feed [20,28]. The percent recovery of virus in these experiments is represented as the time 0 time point [20,28]. After each incubation period, 10 mL of eluent was added to each sample, mixed thoroughly, and then centrifuged to remove organic matter/debris. The supernatants were serially diluted10-fold in maintenance medium to determine the amount of surviving virus, if any. For titration, supernatant dilutions were inoculated into Vero-81and ST monolayers in 96-well microtiter plates (Nunc, Rochester, NY) using 100μL/well. Three wells were used per dilution. Inoculated cells were incubated at 37°C under 5% CO_2_ until CPE appeared. Virus titers were calculated as TCID_50_/mL using the Karber method [29]. The highest dilution showing CPE was considered the end point.

### Statistical analysis

Virus concentration data (log TCID_50_/mL) was modeled by GInaFiT [30]. Weibull distribution model was used to describe inactivation patterns because it provided a better fit of our data, which showed a non-linear rate of inactivation. Because the survival data of the viruses is more accurately matches a Weibull distribution, Mafart et al. (2001) developed the following Weibullian equation [31]:

$$Log\left(N\right)=Log\left(N_{o}\right)-{\left(\frac{t}{\delta }\right)}^{n}$$ 1

In this equation, *N* is the surviving virus after the treatment expressed as (log TCID_50_/mL), *N*_*0*_ is the initial virus titer (log TCID_50_/mL), Delta (*δ)* is the time of the first logarithm decline for the virus titer population (days), and *n* is the shape parameter.

Three valid replicates were used to evaluate how well the model fit with the experimental data by calculating the adjusted R^2^ (Adj. R^2^) as follows:

$$Adj.\;R^{2}=\left[1-\frac{\left(m-1\right)(1-\frac{SSQ\;regression}{SSQ\;total}}{m-j}\right]$$ 2

where *m* is the number of observations, *j* is the number of model parameters, and *SSQ* is the sum of squares.

An ANOVA test using the mixed procedure of SAS (SAS Inst., Cary, NC) was used to compare differences among delta values. Least squared means with Tukey adjustment were used to determine differences among treatment means; *P* < 0.05 was considered to be significantly different. The fixed effect was the feed ingredient being analyzed. Results for each virus were analyzed independently. A correlation analysis using the corr procedure of SAS (SAS Inst., Cary, NC) was done to determine potential associations between feed and feed ingredient chemical composition and the delta values for complete feed or each ingredient.

## Results

In general, all virus inactivation data over time were non-linear, with tails and shoulders in the curves. Because of this, the Weibull model was generally a better representation of the data, resulting in greater Adj. R^2^ values compared with those obtained with the log linear model. Delta values from the Weibull model (time necessary to reduce virus concentration in 1 log) were compared among feed and feed ingredient samples for PEDV, PDCoV and TGEV to characterize virus inactivation kinetics (Table 2). There were no differences in delta values for PEDV among the different feed or ingredient samples. Delta values were greater for PDCoV in soybean meal (42.04 days) and corn (25.60 days) samples compared with the other ingredients, indicating lower inactivation kinetics of PDCoV when incubated in soybean meal and corn. A similar trend was observed for TGEV, where soybean meal had a greater delta value (41.94 days) compared with that observed for the other ingredients.

**Table 2:** Comparison between delta values in feed ingredients for Porcine epidemic diarrhea virus, Porcine Delta Coronavirus, and Transmissible Gastroenteritis Virus.

Ingredient	PEDV		PDCoV		TGEV	
	Delta	Adj. R^2^	Delta^1^	Adj. R^2^	Delta^1^	Adj. R^2^
Feed	1.12 ±0.83	0.76	2.29 ±0.61^a^	0.91	3.20±2.97^a^	0.79
Plasma	1.14 ±1.25	0.72	3.25 ±2.30^a^	0.89	19.18 ±4.26^a^	0.90
Meat Meal	3.87 ±2.16	0.57	2.82 ±1.41^a^	0.79	1.04 ±1.70^a^	0.94
Meat and Bone meal	4.90 ±3.90	0.80	6.22 ±0.87^a^	0.89	0.99 ±0.92^a^	0.95
Blood meal	2.84 ±0.73	0.87	1.23 ±1.31^a^	0.69	2.15 ±0.96^a^	0.91
Corn	2.25 ±1.60	0.83	25.60 ±1.37^b^	0.88	11.78 ±16.77^a^	0.59
Soybean meal	7.50 ±4.61	0.90	42.04 ±14.00^c^	0.50	41.94 ±19.81^b^	0.41
Low oil DDGS	0.70 ±1.07	0.88	6.23 ±2.23^a^	0.92	1.04 ±1.44^a^	0.88
Medium oil DDGS	7.32 ±5.90	0.77	3.76 ±2.43^a^	0.93	1.66 ±1.16^a^	0.85
High oil DDGS	0.56 ±0.49	0.72	8.80 ±4.40^a^	0.90	0.78 ±0.95^a^	0.81
**SEM**	**1.70**		**2.82**		**4.24**	
**P value**	**0.0586**		**0.0001**		**0.0001**	

^1^Different letters within the same column differ (*P* < 0.05)

At the conclusion of the 56-day incubation period, the titers for all PEDV samples were 0.50 log TCID_50_/mL, except for soybean meal, which was greater at 0.83 log TCID_50_/mL (Table 3). For PDCoV, day 0 titers were greater than those for PEDV, and ranged from 3.72 to 6.61 log TCID_50_/mL (Table 4). After the 56-day storage period, the greatest log reduction for PDCoV was observed in low oil DDGS (5.78) and high oil DDGS (5.45). The greatest virus survival occurred in soybean meal, with only 1.44 log reduction. A similar trend was observed for TGEV survival, where samples inoculated with TGEV had the greatest initial virus titers ranging from 3.51 to 7.17 log TCID_50_/mL (Table 5). After the 56-day storage, the greatest log reduction was achieved in high oil DDGS (6.56) and medium oil DDGS (5.67) samples. Similar to PDCoV, the least log reduction was observed in soybean meal, with only 2.22 log reduction in the initial virus concentration.

**Table 3:** Porcine epidemic diarrhea virus titer during the 56 day incubation period in feed ingredients.

Time (days)	PEDV Virus titer (log TCID_50_/mL)									
	Feed	SDPP^1^	Meat Meal	Meat Bone Meal	Blood Meal	Soybean Meal	Corn	Low oil DDGS^2^	Medium oil DDGS^2^	High oil DDGS^2^
0	4.28	2.06	2.83	3.06	3.84	3.50	2.84	3.51	2.51	3.94
1	2.51	1.51	2.17	2.28	2.95	3.84	2.51	1.73	1.51	2.18
3	1.51	0.50	1.17	1.94	2.39	3.28	2.40	1.62	1.28	1.62
7	1.40	0.50	1.51	1.72	1.61	2.83	1.51	1.72	1.51	1.51
14	0.61	1.06	1.50	1.51	1.51	2.06	0.50	1.51	1.51	1.51
21	0.50	0.83	1.62	1.51	1.51	1.62	0.50	1.40	1.17	1.51
28	1.17	0.50	1.51	1.51	1.51	1.51	0.50	0.61	0.72	1.51
35	0.50	0.50	1.51	1.51	1.51	1.40	0.50	0.50	0.50	1.51
42	0.50	0.50	1.51	1.51	0.50	0.50	0.50	0.50	0.50	1.51
49	0.50	0.50	0.50	0.50	0.50	0.72	0.50	0.50	0.50	1.51
56	0.50	0.50	0.50	0.50	0.50	0.83	0.50	0.50	0.50	0.50
**Log Reduction**										
7	2.89	1.56	1.32	1.33	2.23	0.67	1.33	1.79	1.00	2.43
14	3.67	1.01	1.33	1.55	2.33	1.45	2.34	2.00	1.00	2.43
28	3.11	1.56	1.32	1.55	2.33	1.99	2.34	2.90	1.79	2.43
42	3.78	1.56	1.32	1.55	3.34	3.00	2.34	3.01	2.01	2.43
56	3.78	1.56	2.33	2.56	3.34	2.67	2.34	3.01	2.01	3.44

^1^ Spray dried porcine plasma

^2^ Dried distillers grains

**Table 4:** Porcine Delta Coronavirus (PDCoV) titer during 56 day incubation in feed ingredients.

Time (days)	PDCoV Virus Titer Log TCID50/mL									
	Feed	SDPP^1^	Meat Meal	Meat Bone Meal	Blood Meal	Soybean Meal	Corn	Low oil DDGS^2^	Medium oil DDGS^2^	High oil DDGS^2^
0	5.51	5.62	5.51	5.72	5.51	6.61	3.72	6.28	5.51	5.95
1	5.28	5.06	5.17	6.17	5.28	4.51	4.73	6.06	5.28	5.61
3	4.40	3.73	4.61	4.29	4.40	5.17	3.51	4.51	4.40	4.73
7	3.17	3.95	3.61	4.95	3.17	5.50	3.61	5.39	3.17	5.28
14	2.73	2.51	2.40	4.06	2.73	5.40	4.40	3.84	2.73	3.73
21	2.51	2.51	3.17	3.51	2.51	5.40	3.51	3.95	2.51	3.84
28	2.51	2.51	3.51	3.51	2.51	5.29	2.84	2.51	2.51	2.51
35	2.62	2.51	2.84	2.61	2.62	5.51	2.40	2.40	2.62	3.29
42	1.28	0.95	2.17	2.51	1.28	5.06	1.51	0.51	1.28	1.06
49	0.50	0.51	1.95	1.95	0.50	5.40	0.51	0.51	0.50	0.51
56	0.50	0.51	1.51	1.51	0.50	5.17	0.51	0.51	0.50	0.51
**Log Reduction**										
7	2.34	1.67	1.89	0.77	2.34	1.11	0.11	0.89	2.34	0.67
14	2.78	3.11	3.11	1.67	2.78	1.22	0.00	2.44	2.78	2.22
28	3.00	3.11	2.00	2.22	3.00	1.33	0.89	3.78	3.00	3.45
42	4.23	4.67	3.33	3.22	4.23	1.55	2.22	5.78	4.23	4.89
56	5.01	5.11	4.00	4.22	5.01	1.44	3.22	5.78	5.01	5.45

^1^ Spray dried porcine plasma

^2^ Dried distillers grains

**Table 5:** Transmissible Gastroenteritis virus (TGEV) titer during 56 day incubation in feed ingredients.

Incubation Period	TGEV Virus titer Log TCID50/mL									
	Feed	SDPP^1^	Meat Meal	Meat Bone Meal	Blood Meal	Soybean Meal	Corn	Low oil DDGS^2^	Medium oil DDGS^2^	High oil DDGS^2^
0	5.61	3.51	6.06	5.28	5.83	7.17	4.51	6.62	6.17	7.06
1	4.50	3.95	4.62	3.51	4.95	4.61	3.17	4.06	3.84	4.06
3	3.40	3.51	3.17	3.29	4.06	5.40	2.06	3.84	3.06	3.06
7	2.72	3.40	2.51	2.51	3.06	4.95	2.51	2.95	2.61	2.51
14	2.51	3.51	2.51	2.40	2.29	4.51	3.61	2.51	2.40	2.51
21	2.51	2.73	1.51	1.51	1.40	5.51	2.18	1.61	2.51	2.40
28	2.51	2.51	1.51	1.51	1.51	4.73	2.29	2.28	2.17	2.51
35	2.51	1.51	1.28	1.51	1.51	5.29	1.95	2.06	1.51	1.84
42	2.51	0.51	0.95	0.61	1.51	4.50	1.61	0.51	0.51	1.72
49	1.06	0.51	0.51	0.51	0.51	5.17	1.51	0.51	0.51	0.51
56	0.51	0.51	0.51	0.51	0.51	4.95	0.51	0.51	0.51	0.51
**Log Reduction**										
7	2.89	0.11	3.56	2.78	2.77	2.22	2.00	3.67	3.56	4.56
14	3.11	0.00	3.56	2.88	3.55	2.67	0.89	4.11	3.78	4.56
28	3.11	1.00	4.56	3.78	4.33	2.44	2.22	4.34	4.00	4.56
42	3.11	3.00	5.11	4.67	4.33	2.67	2.89	6.11	5.67	5.34
56	5.11	3.00	5.56	4.78	5.33	2.22	4.00	6.11	5.67	6.56

^1^ Spray dried porcine plasma

^2^ Dried distillers grains

A Pearson correlation was performed to examine associations between ingredient chemical composition and virus survival. There was a moderate positive correlation for TGEV (r = 0.41, *P* = 0.02) and PDCoV (r = 0.49, *P* = 0.01) survival with moisture content. This suggests that increased moisture content may lead to increased virus survival, but this correlation was not observed for PEDV (Table 6). Furthermore, there was a moderate negative correlation between TGEV survival and ether extract content (r = -0.51, P = 0.01), suggesting increased ether extract content may reduce virus survival time, but this correlation was not observed for PEDV or PDCoV (Table 6).

**Table 6:** Pearsons correlation coefficients (r), among feed ingredient composition and time necessary for first log reduction in transmissible gastroenteritis virus (TGEV), Porcine Epidemic Diarrhea Virus (PEDV), Porcine Delta Coronavirus (PDCoV) concentration.

Composition Correlation	Virus		
	TGEV	PEDV	PDCoV
**Moisture, r**	0.41	-0.05	0.48
**Moisture, *P*-Value**	0.03	0.81	0.01
**Fat, r**	-0.51	-0.01	-0.31
**Fat, *P*-Value**	0.01	0.99	0.10
**Crude Protein, r**	0.07	0.08	-0.31
**Crude Protein, *P*-Value**	0.71	0.69	0.10
**Ash, r**	-0.19	0.14	-0.23
**Ash, P-Value**	0.34	0.46	0.22
**Crude fiber, r**	-0.17	0.02	0.11
**Crude fiber, *P*-Value**	0.38	0.90	0.58

## Discussion

Previous research demonstrated that the survival of PEDV varies among feed ingredients, and it appears to survive for the longest time in soybean meal [23]. These previous experiments compared the virus survival at stored external temperatures [23]. No studies have been conducted to evaluate PDCoV and TGEV survival among various types of feed ingredients. Similarly, no studies have been conducted to evaluate the survival of PEDV, PDCoV, and TGEV among various types of feed ingredients at controlled temperatures. The hypothesis for our experiment was that virus survival would be associated with the chemical composition of each ingredient. One of these components is the pH of the feed ingredients. Porcine epidemic diarrhea virus is stable at a pH between 6.5 to 7.5 [32], and results from a previous experiment showed that the virus is more sensitive to heat treatments at a greater pH of 10.2 compared to 7.2 [33]. These findings suggest that virus survival in ingredients that have a pH outside of this favorable range may be lower. When comparing the moisture content of chemical composition, previous experiments have demonstrated that PEDV survives up to 28 days in wet complete feed, but only 7 days in dry complete feed [34]. Transmissible Gastroenteritis Virus was used as a control in this experiment and demonstrated the same behavior, with higher survival in wet feed [34]. These data lead to the hypothesis that higher moisture ingredients will also have a higher survival of PEDV, PDCoV, and TGEV.

In our experiment, a variation in virus survival was observed among various ingredients in all three viruses right away at day 0. The titer of the virus inoculated into the sample was identical for all ingredients with an average titer of 3.2x10^4^ for PEDV, 1.5x10^6^ for PDCoV, and 6.8x10^6^ for TGEV. Though the initial inoculum had identical titers, an immediate difference was observed in virus concentration among ingredients in all three viruses. For example, titer of PEDV dropped to 2.06 log TCID_50_/mL in SDPP from the initial 4 logs of virus that was inoculated into the sample. This indicates that some ingredients had an immediate effect on virus survival that caused a decrease before the incubation period even started. This immediate variation in virus survival was also observed in another experiment measuring on the survival of PEDV in various feed ingredients, with CT values ranging from 16 to 36 on the first day of incubation despite being inoculated with the same virus inoculum of a CT value of 16.3 [23]. This immediate variation in virus concentration could also indicate differences in percent recovery of virus using our modified elution method between the different viruses and feed ingredients. To account for some of this potential variation in elution potential, the determination of the delta value and overall log reductions used a comparison to the day 0 time point for that ingredient, when the virus was placed in the feed sample and immediately eluted. The day 0 time point accounts for the decrease in virus titer because of the elution process, but since it was immediately eluted there will be no decrease in virus titer because of storage in the ingredient. This accurately models the inactivation of each virus over time, even if there are differences in the percent virus recovery among feed ingredients or different viruses. In addition to variation in elution between feed ingredients, there is also a likely difference in elution between the three viruses. To account for this variation, all data for each virus was analyzed separately and results from this experiment cannot be used to make comparisons between the three viruses. Instead, comparisons should be made for each virus on its own between the various feed ingredients evaluated.

In addition to comparing initial virus concentration, there is also a variation in overall log reduction among feed ingredients for each of the three viruses. Previous research suggests a dose of 5.6 x 10^1^ TCID_50_/mL, or 1.7 log TCID_50_/mL, of PEDV is necessary to cause an active infection in a feed matrix [17]. Using our starting virus titer of 4.5 log TCID_50_/mL, this threshold of 1.7 log TCID_50_/mL was reached at 3 days in all of the ingredients except meat and bone meal, blood meal, soybean meal, and corn. At 14 days, the virus concentration was below this limit in all of the ingredients except soybean meal. It was not until 21 days until the virus concentration in soybean meal dropped below the proposed 1.7 log TCID_50_/mL threshold. An infectious dose for PDCoV and TGEV has not yet been determined in a feed matrix, but soybean meal also had the highest survival for both viruses with a virus concentration of 5.17 log TCID_50_/mL of PDCoV and 4.95 log TCID_50_/mL of TGEV even after 56 days of incubation.

When comparing the inactivation kinetics of each virus, there is a similar trend for soybean meal to have the greatest virus survival. With such high survival of all three viruses in soybean meal, a correlation was done to determine what feed characteristic might cause this high survival. As stated in our hypothesis, moisture was one of the variables believed to have an impact on virus survival. This was somewhat confirmed in the correlation which determined a moderate positive relationship between PDCoV (r = 0.4823, *P* < 0.05) and TGEV (r = 0.4128, *P* < 0.05). This means that as predicted, as moisture content increases, so does virus survival. However, the same relationship was not observed in PEDV survival. Contradicting our hypothesis, there was no correlation between pH and virus survival. This is probably because our experiment was done in dry feed ingredients. Other experiments evaluating the impact of pH on virus survival that have shown an effect were performed in liquid samples [33]. In addition to moisture, there was also a significant negative correlation between ether content and TGEV survival (-0.5067, *P* < 0.05). This correlation was unexpected and only observed in TGEV survival.

One of the major limitations to this experiment was the addition of liquid media necessary to inoculate the feed samples with virus. The addition of 1mL of liquid media to 5 gram aliquots of feed or ingredient greatly increased the moisture content of the sample. As is evident from our results, moisture plays a significant role in virus survival and hence a change in moisture content will alter virus survival kinetics as opposed to that observed in completely dry feed. Though the moisture content was altered, the same amount of moisture was added to each feed ingredient making it possible to still compare virus survival among various ingredients. A second limitation to our experiment was the low virus titers used in the PEDV experiment. The concentration of PEDV used in the experiments was at the maximum titer that could be obtained for this virus by using the presented methods. Though the concentration of PEDV in the sample could be increase by adding more inoculum to each vial, this would also increase the moisture in the sample and contribute to the limitations previously stated. These low titers limited the amount of reduction that could occur in virus concentration, which then limited the variation we could observe among feed ingredients. We hypothesize that if we were working with higher concentrations of virus, we would have observed a statistically higher delta value for PEDV survival in soybean meal, as opposed to just numerically higher value. Having higher delta values for PEDV could then minimize the difference observed between PEDV and the other two coronaviruses. The second problem with the low virus titers is that they may not represent the virus titers observed in the field. A study measuring the residual amount of PEDV in a contaminated feed bin estimated these CT values between 19.5 and 22.2 [16]. This amount of virus is equivalent to 8.9 to 9.2 log copies of RNA/g based on a calibration curve obtained from the University of Minnesota [35]. With our low titers of virus, it was impossible for us to show an 8 to 9 log reduction in virus concentration that would be necessary to inactivate PEDV in a realistic scenario. Despite the low virus titers producing challenges in the comparison between PEDV inactivation and TGEV or PDCoV inactivation, the initial low virus titer was similar between ingredients inoculated with PEDV. This indicates that a comparison can still be made between the inactivation of PEDV in the evaluated feed ingredients.

## Conclusions

Overall, we conclude that the survival of PEDV, PDCoV, and TGEV varies based on feed ingredient. Out of the feed ingredients analyzed, soybean meal had greatest delta value in all three viruses, along with the highest concentration of remaining virus after 56 days of incubation. These results validated previous research demonstrating extended coronavirus survival in soybean meal. These results also suggest that soybean meal may be a risk factor for virus transmission if contamination occurs, but the likelihood of contamination has yet to be evaluated.
